# 

**Published:** 1888-09

**Authors:** 


					SsfcCHTfcCM.BKK, !???.
THE
AMERICAN JOURNAL
?HOO F**0'
DENTAL SCIENCE.
EDITED BY
F. J. S. SOMAS, M. D., D. D. S.
UOLt. 22
THIRD SERIES.
f*D. B.
BALTIMORE:
SNOWDEN &. COWMAN.
LONDON:
TRUBNER& CO., &O PATERNOSTER ROW.
:PMKBLE IN 1DYMCE.
CPITBTJSRED MONTHLY.:
$2.50 PER KNNUM.:

				

